# Supplementary material for: MSNovelist: de novo structure generation from mass spectra
Source: Nat Methods. 2022 May 30;19(7):865–70. doi: 10.1038/s41592-022-01486-3 (PMC9262714; doi:10.1038/s41592-022-01486-3)
Supplement: Supplementary file 1 — Supplementary Figs. 1–8, Supplementary Algorithms 1 and 2 and Supplementary Tables 1–13 [file 41592_2022_1486_MOESM1_ESM.pdf]

nature portfolio

https://doi.org/10.1038/s41592-022-01486-3

Supplementary information

MSNovelist: de novo structure generation from mass spectra

In the format provided by the authors and unedited

MSNovelist: *De novo* structure generation from mass spectra

Michael A. Stravs[1‡], Kai Dührkop[2], Sebastian Böcker[2], Nicola Zamboni[1*]

[1] Institute of Molecular Systems Biology, ETH Zürich, CH-8092 Zürich, Switzerland

[2] Institut für Informatik, Friedrich-Schiller-Universität Jena, D-07743 Jena, Germany

[‡] stravs@imsb.biol.ethz.ch, * corresponding author, zamboni@imsb.biol.ethz.ch

Supplementary Information

Supplementary Figure 1: Model architecture. *The encoder generates a reduced-dimensionality representation z from fingerprint* x^M *and molecular formula* x^F*, and computes initial states for the decoder LSTM network. The decoder is composed from a recurrent neural network (Dec) which predicts an output token per timestep from the preceding token, the latent representation z, and an auxiliary vector v that counts remaining elements and open brackets in the SMILES code (Hint). An auxiliary LSTM network (H_Count) estimates hydrogen atom counts per token for use in the element counter. Concatenations are omitted in the scheme.*

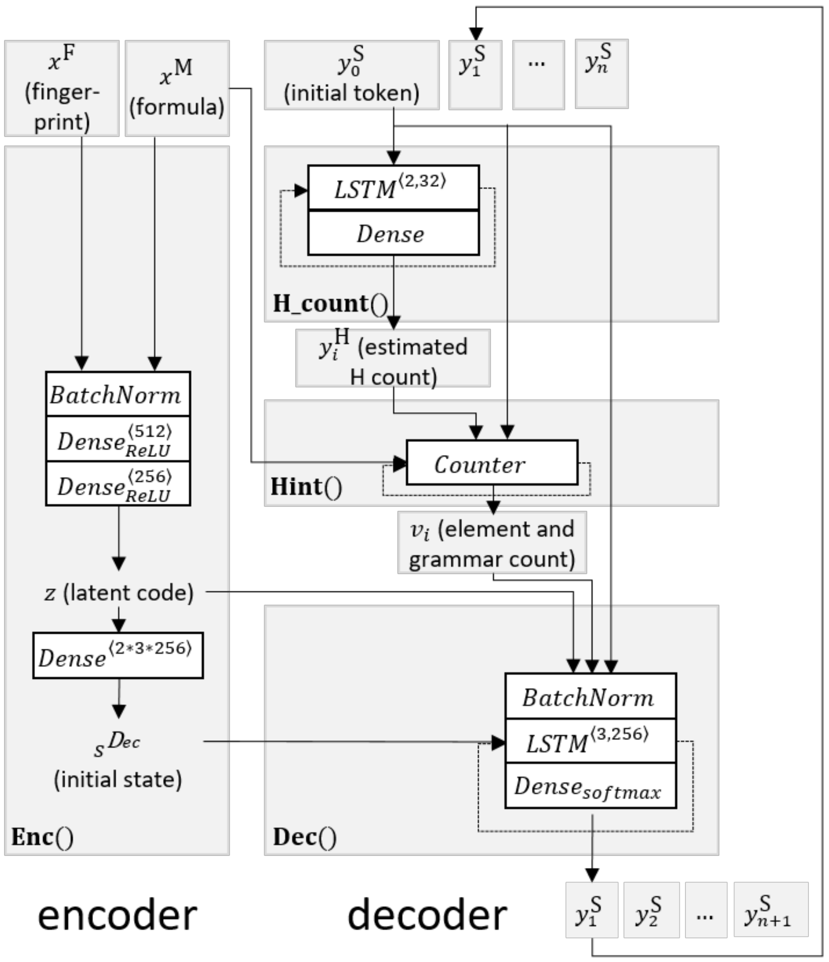

Supplementary Figure 2: Seven randomly chosen incorrect MSNovelist predictions from the GNPS dataset. *Structures 4-10a: de novo prediction; structures 4-10b: correct result. Red color marks sites predicted incorrectly by the model (or the entire molecule if the prediction was completely wrong), blue color marks the corresponding correct alternative.*

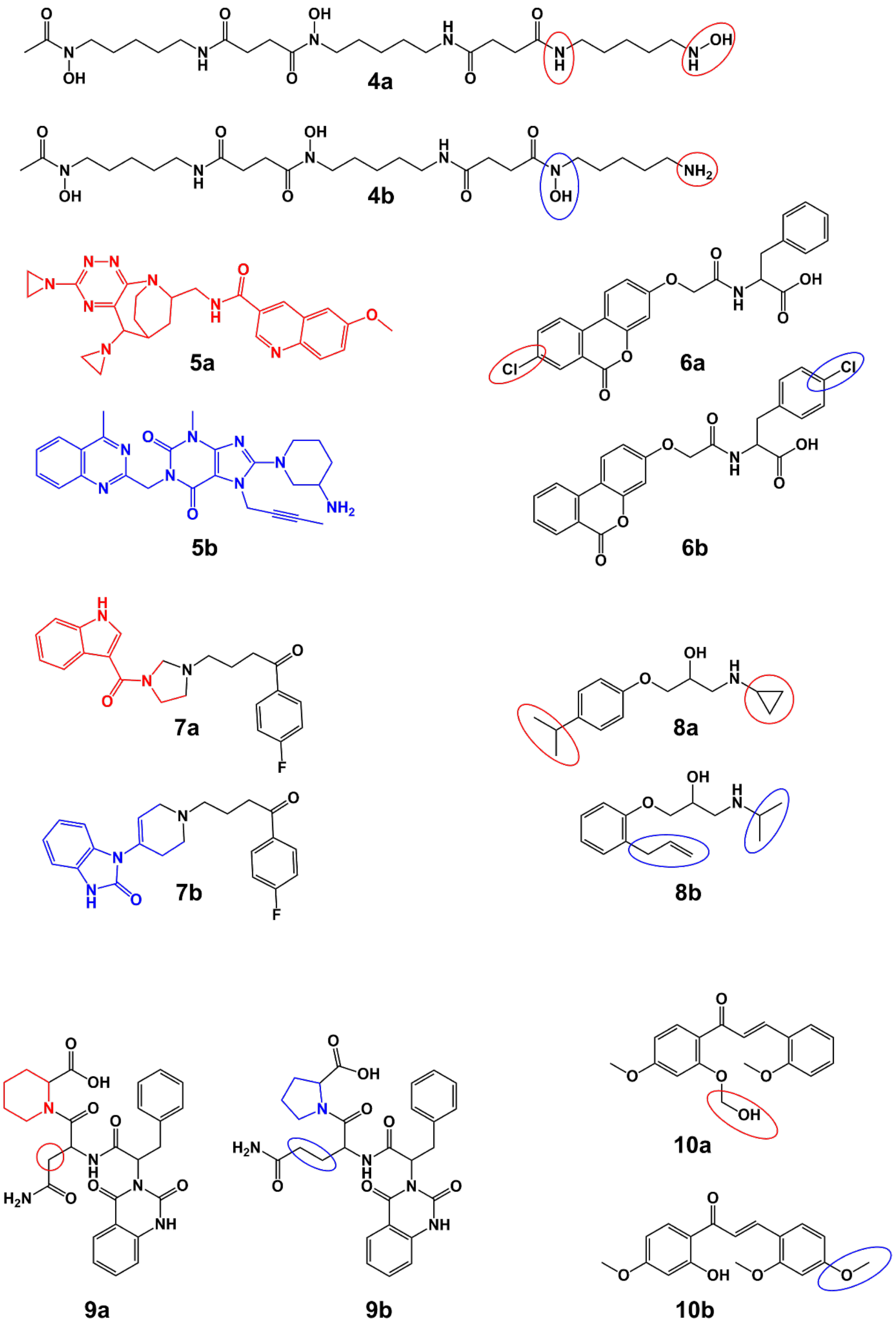

Supplementary Figure 3: Model evaluation for CASMI dataset. *Rank of correct structure in results for MSNovelist prediction (blue), and naïve generation (orange), generated with top-128 (solid) or top-16 beam search (dashed), with comparison to database search (SIRIUS 4.4.29 on PubChem; green). a), b): Candidates reranked by ModPlatt score; c), d): candidates ordered by raw score (model probability). a), c): CASMI dataset ($n = 127$). b), d): CASMI-top1 dataset ($n = 43$). Note that beam search with $k = 16$ is not identical to selecting the top-16 candidates from a beam search with $k = 128$, leading to small differences between non-reranked top-128 and top-16 results.*

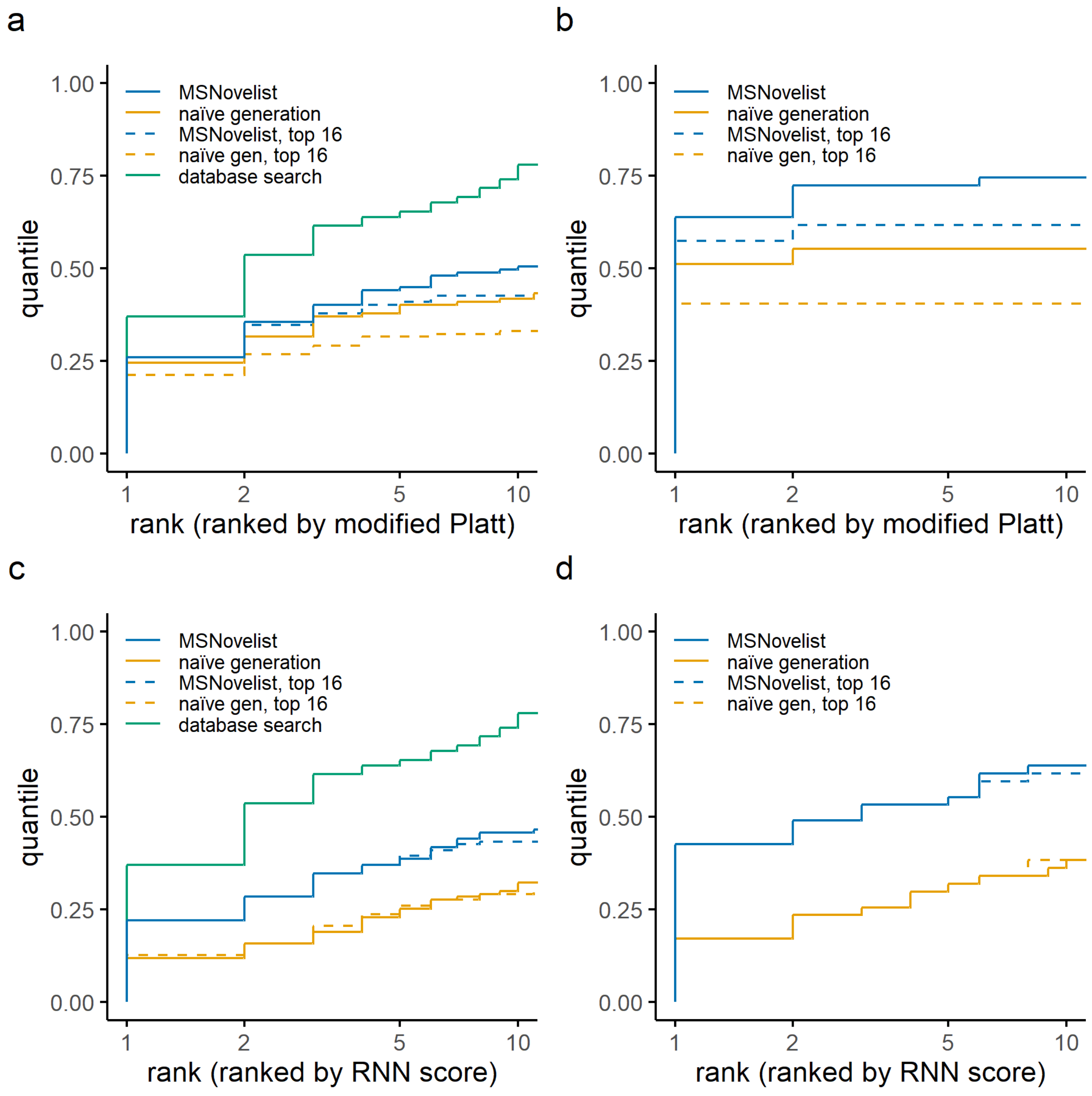

Supplementary Figure 4: Validity of generated SMILES for MSNovelist model and naïve generation *comparing full versus partial models, for GNPS dataset ($n = 3863$). a) Histogram of the fraction of valid SMILES per instance for full MSNovelist model (blue), MSNovelist model without hydrogen counting (green), and MSNovelist model without hydrogen counting and formula/grammar hinting (green). b) Histogram of the fraction of SMILES with correct molecular formula (versus all generated SMILES, including invalid SMILES) for full MSNovelist model, MSNovelist model without hydrogen counting, and MSNovelist model without hydrogen counting and formula/grammar hinting. c) Histogram of the fraction of valid SMILES per instance for full naïve model (blue) and naïve model without hydrogen counting (green). d) Histogram of the fraction of SMILES with correct molecular formula for full naïve model, and naïve model without hydrogen counting. Note: Naïve model with no hinting is not shown, since it generated $\sim$ 100% valid SMILES but $\sim$ 0% structures with correct formula, as expected (see Supplementary Table 1). The full model with MF and Hcount generates the most SMILES with a correct MF. In contrast, it generates the least valid SMILES overall, as its probabilities trade off achieving the correct hypothetical MF versus matching valid SMILES grammar. The model without MF and Hcount generates the most valid SMILES but the least SMILES with correct MF, as its probabilities only implicitly consider the correct target MF.*

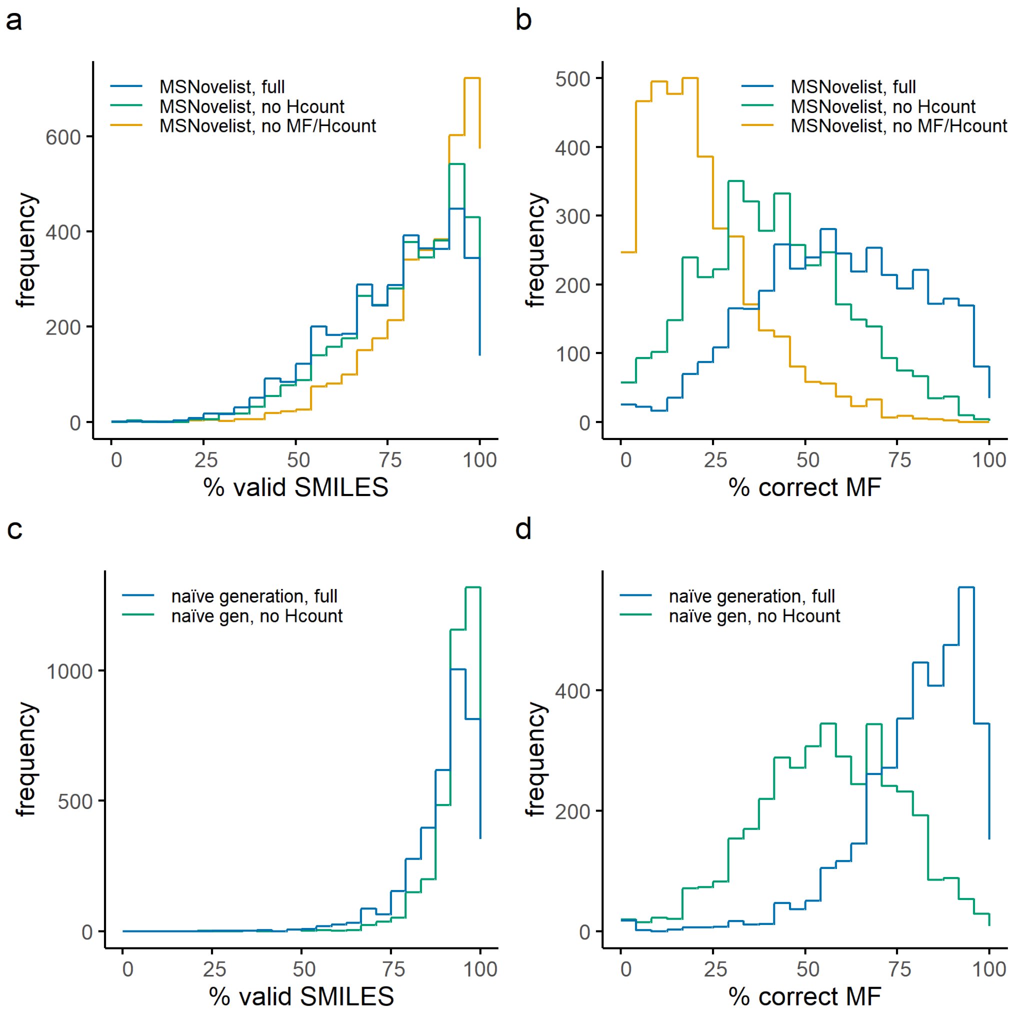

***Supplementary Figure 5: Evaluation of de novo model with and without formula hinting** and hydrogen count, for GNPS dataset (n = 3863). a) Rank of correct structure in results for full de novo model (blue), de novo model without hydrogen counting (green), and de novo model without hydrogen counting and formula/grammar hinting (orange). b) ModPlatt score of top candidate (topscore) ranked by ModPlatt score, for full MSNovelist model, MSNovelist model without hydrogen counting, and MSNovelist without hydrogen counting and formula/grammar hinting, versus best candidate in training set (light blue). c) Tanimoto similarity of best incorrect candidate to correct structure (topsim) for full MSNovelist model, MSNovelist model without hydrogen counting, and MSNovelist model without hydrogen counting and formula/grammar hinting, versus best candidate in training set. d), e), f): idem, ordered by raw score (model probability); versus random choice from training set (red). Note that the model without MF and/or Hcount appears to perform better when ranking by RNN score (d). Since the evaluation takes into account only valid molecules with correct molecular formula, this ranking omits the invalid candidates generated by those models and artificially appears better. A residual difference may exist because the simplified model probabilities do not need to consider formula correctness.*

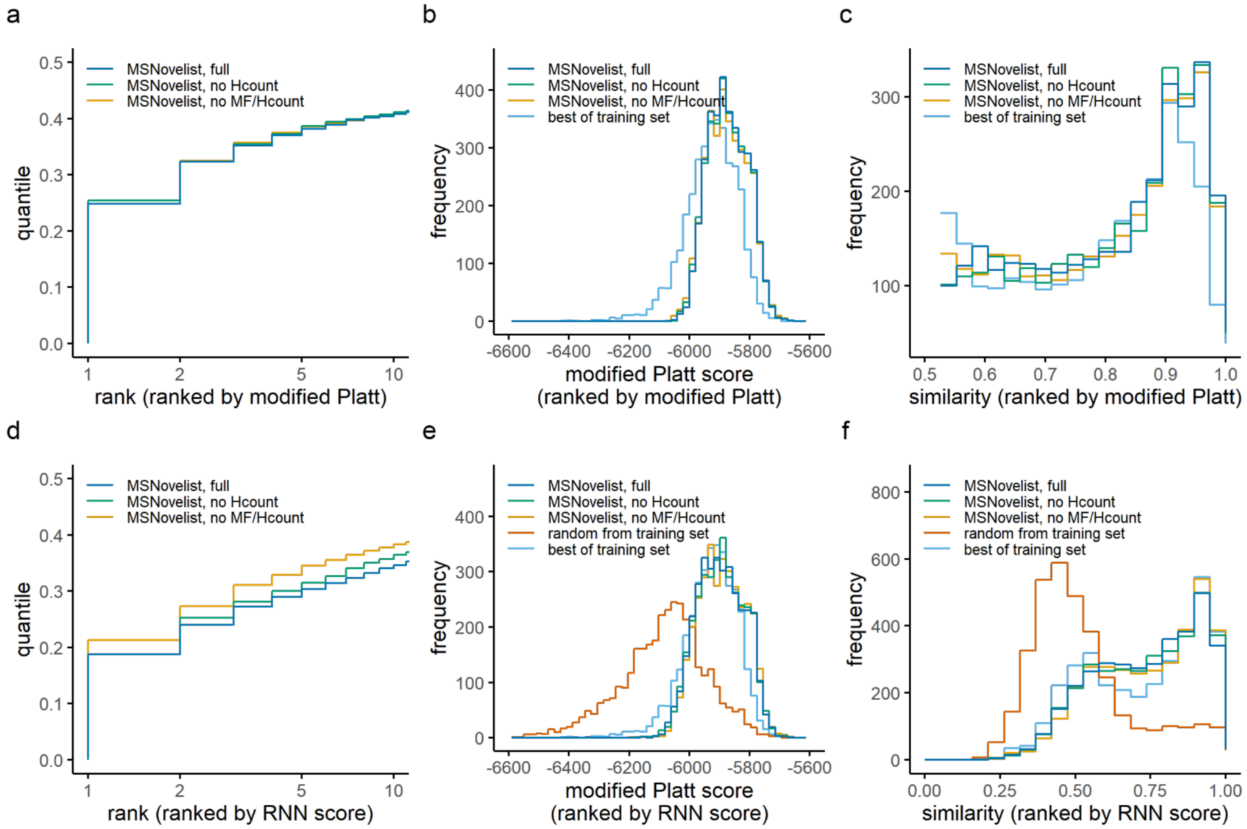

***Supplementary Figure 6: Evaluation of de novo generation versus naïve generation** for top-128 and top-16 beam search, for GNPS dataset ($n = 3863$). a) Rank of correct structure in results for de novo prediction (blue), and naïve generation (orange), generated with $k = 128$ (solid) or $k = 16$ (dotted). b) ModPlatt score of top candidate (topscore) ranked by ModPlatt score, for MSNovelist prediction and naïve generation, with $k = 128$ or $k = 16$, versus best candidate from training set (light blue). c) Tanimoto similarity of best incorrect candidate to correct structure (topsim) for MSNovelist prediction and naïve generation, with $k = 128$ or $k = 16$, versus best candidate from training set. d), e), f): idem, ordered by raw score (model probability); versus random choice from training set (red).*

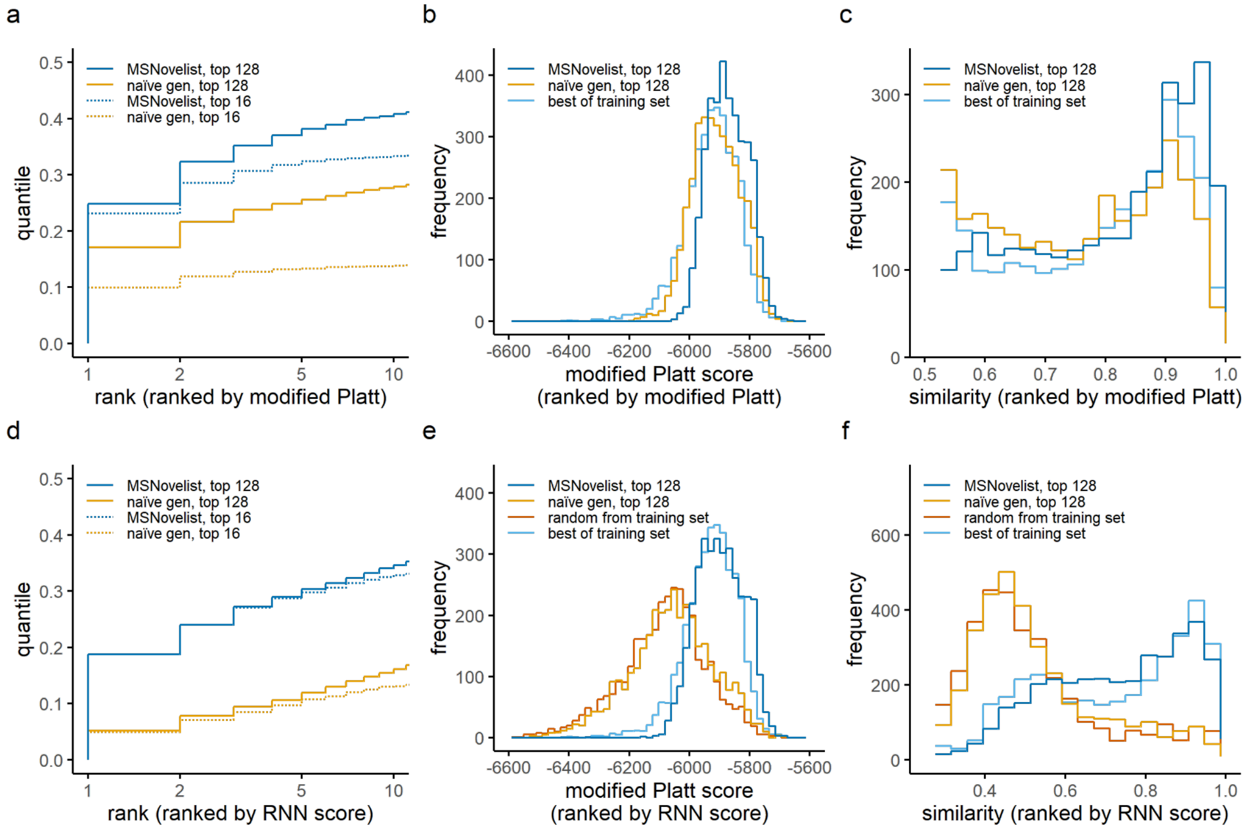

Supplementary Figure 7: Proposed fragmentation for structure 377a.

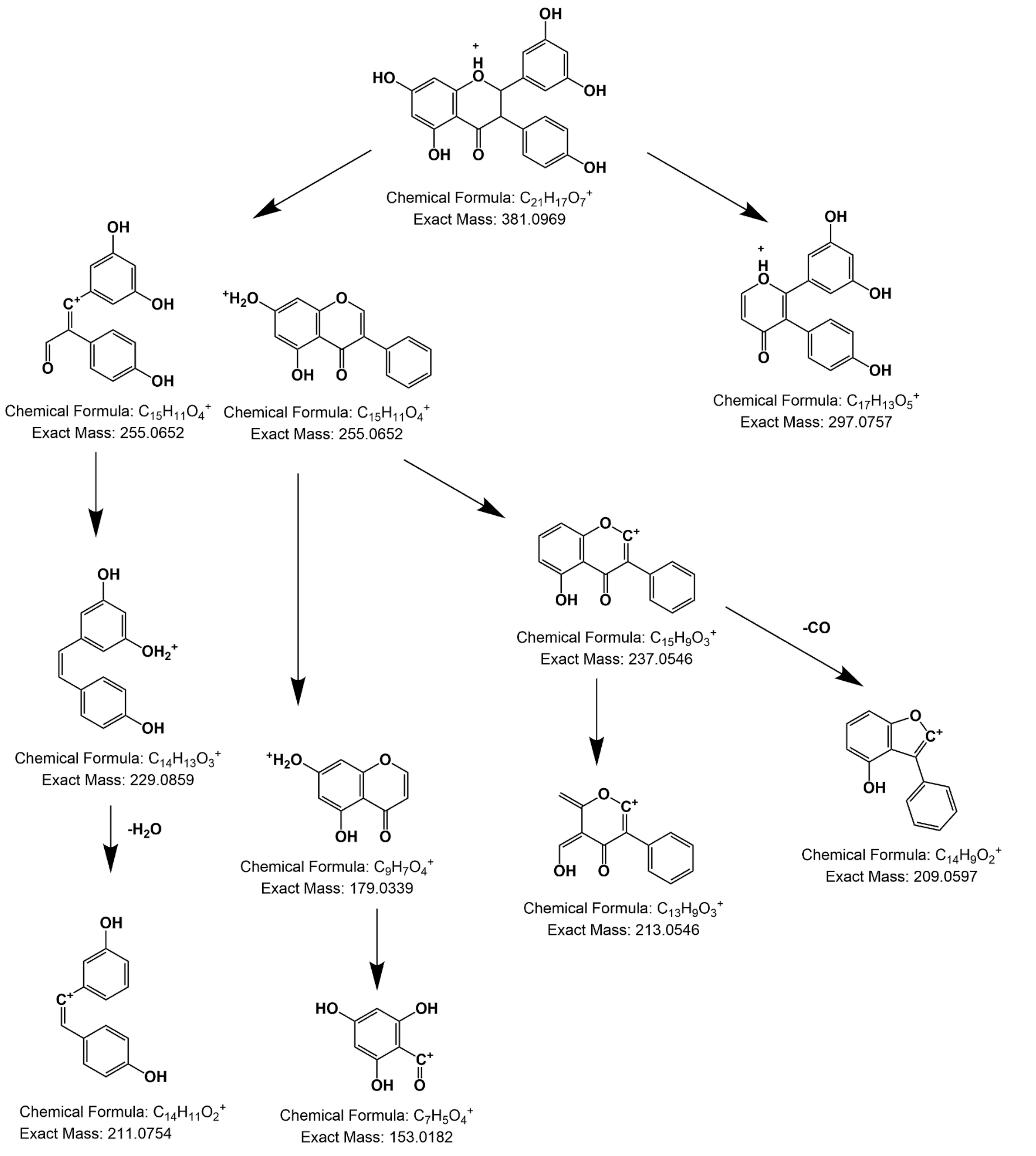

Supplementary Figure 8: ***Scores of best MSNovelist candidates versus best database scores (extended dataset)***. *Regular line: 1:1 line; dashed line:*$ModPlatt_{MSNovelist} = ModPlatt_{DB} + 50$*; labels: spectrum ID. The dataset was processed analogously to the original dataset without a limitation to m/z < 500. Of 667 spectra, 263 obtained a molecular formula with high confidence. 29 structure predictions were identical between MSNovelist and database; 179 MSNovelist predictions scored higher, and 55 database predictions scored higher. Five additional instances crossed the threshold of* $ModPlatt_{MSNovelist} > ModPlatt_{DB} + 50$ *(see Supplementary Table S5). The de novo candidates explained the spectrum peaks better (2 instances) or equally well (3) as the database candidates. We note that feature 467 is not recovered using the full dataset; it was assigned a different formula since ZODIAC formula annotation is dependent on the entire dataset.*

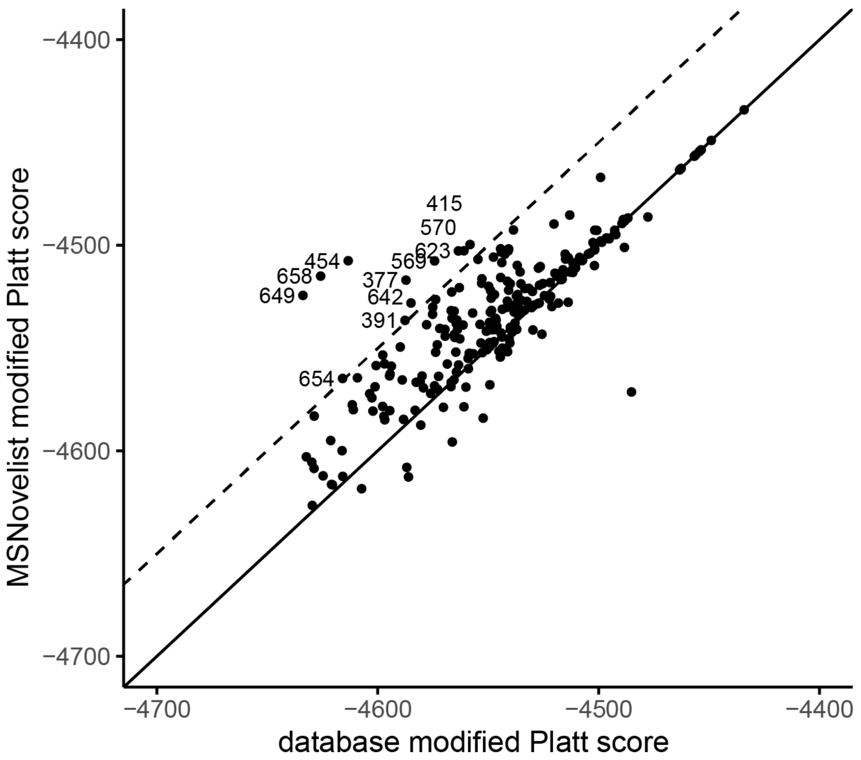

Supplementary Algorithm 1: Fingerprint simulation

input : $x_{struct} = \{0,1\}^n$: struct-FP
input : Dataset $D = \left(x'_{struct} \in \{0,1\}^n, x'_{spec} \in \mathbb{R}^n\right)^{(i)}$:
tuples of struct-FP and CV-spec-FP in CV-spec-FP dataset
output : $x_{sim} \in \mathbb{R}^n$: sim-FP
for $i \leftarrow 1$ **to** n **do**
  $D' \leftarrow$ subset D: $x'_{struct_i} = x_{struct_i}$;
  $x^*_{struct}, x^*_{spec} \leftarrow$ sample one item from D';
  $x_{sim_i} \leftarrow x^*_{spec_i} + jitter$;
end

Supplementary Algorithm 2: Beam search

input : $\hat{P}\left(y^{S}{}_{i+1} = j\right), s_{i+1} \middle| y^{S}{}_{1..i}, s_i, z$: sequence model
input : s_0: initial state
input : z: context vector
$sequences^{(1..k)} \leftarrow$ initial token;
$scores^{(1..k)} \leftarrow 0$;
$states^{(1..k)} \leftarrow s_0$;
$finalsequences \leftarrow \emptyset$;
$finalscores \leftarrow \emptyset$;
for $i \in (1..l)$ **do**
  $states^{(j\in 1..k)} \leftarrow s_{i+1} \left| y^{S}{}_{1..i} = sequences^{(j)}, s_i = states^{(j)}, z \right.$;
  $candidatesequences^{(j\in 1..k\times t)} \leftarrow$ expand all $sequences^{(1..k)}$
  with all possible $tokens^{(1..t)}$;
  $tokenscores^{(j\in 1..k\times t)} \leftarrow log\left(\hat{P}\left(y^{S}{}_{i+1} = j\right)\right) \Bigg| \begin{matrix} y^{S}{}_{1..i} = candidatesequences^{(j)}, \\ s_i = states^{(j \bmod t)}, z \end{matrix}$;
  $candidatescores^{(j\in 1..k\times t)} \leftarrow scores^{(j \bmod t)} + tokenscores$;
  set scores for padding character to $-\infty$;
  $topsequences \leftarrow \mathrm{argtop}_k(candidatescores)$;
  $sequences = candidatesequences^{(topsequences)}$;
  $scores \leftarrow candidatescores^{(topsequences)}$;
  $states \leftarrow states^{(topsequences \bmod t)}$;
  for j **where** the last token of $sequences^{(j)}$ is termination token **do**
    add $sequences^{(j)}$ to $finishedsequences$
    add $scores^{(j)}$ to $finishedscores$
  end
end
$topfinalsequences \leftarrow \mathrm{argtop}_k(finalscores)$;
$selectedfinalsequences \leftarrow finalsequences^{(topfinalsequences)}$;
$selectedfinalscores \leftarrow finalscores^{(topfinalsequences)}$;
return : $selectedfinalsequences, selectedfinalscores$

Supplementary Tables 1-13: see "SupplementaryTables.xlsx"

Supplementary Table 1: Evaluation results for dataset GNPS.

Supplementary Table 2: Evaluation results for dataset GNPS-OK.

Supplementary Table 3: Evaluation results for dataset CASMI.

Supplementary Table 4: Evaluation results for dataset CASMI-OK.

Supplementary Table 5: De novo annotation of bryophyte metabolites, summary.

Supplementary Table 6: MS2 spectrum of feature 391 and spectrum interpretation for proposed structures 391a (MSNovelist prediction) and 391b (database search).

Supplementary Table 7: Additional information for feature 377.

Supplementary Table 8: MS2 spectrum of feature 391 and spectrum interpretation for proposed structures 391a (MSNovelist prediction) and 391b (database search).

Supplementary Table 9: MS2 spectrum of feature 415 and spectrum interpretation for proposed structures 415a (MSNovelist prediction) and 415b (database search).

Supplementary Table 10: MS2 spectrum of feature 454 and spectrum interpretation for proposed structures 454a (MSNovelist prediction) and 454b (database search).

Supplementary Table 11: MS2 spectrum of feature 467 and spectrum interpretation for proposed structures 467a (MSNovelist prediction) and 467b (database search).

Supplementary Table 12: MS2 spectrum of feature 569 and spectrum interpretation for proposed structures 569a (MSNovelist prediction) and 569b (database search).

Supplementary Table 13: MS2 spectrum of feature 570 and spectrum interpretation for proposed structures 570a (MSNovelist prediction) and 570b (database search).
