# Supplementary material for: MSNovelist: de novo structure generation from mass spectra
Source: Nat Methods. 2022 May 30;19(7):865–70. doi: 10.1038/s41592-022-01486-3 (PMC9262714; doi:10.1038/s41592-022-01486-3)
Supplement: Supplementary file 2 — Reporting Summary [file 41592_2022_1486_MOESM2_ESM.pdf]

nature portfolio

Corresponding author(s): Nicola Zamboni

Last updated by author(s): Feb 26, 2022

Reporting Summary

Nature Portfolio wishes to improve the reproducibility of the work that we publish. This form provides structure for consistency and transparency in reporting. For further information on Nature Portfolio policies, see our Editorial Policies and the Editorial Policy Checklist.

Statistics

For all statistical analyses, confirm that the following items are present in the figure legend, table legend, main text, or Methods section.

n/a	Confirmed	
☐	☒	The exact sample size (n) for each experimental group/condition, given as a discrete number and unit of measurement
☒	☐	A statement on whether measurements were taken from distinct samples or whether the same sample was measured repeatedly
☒	☐	The statistical test(s) used AND whether they are one- or two-sided<br>*Only common tests should be described solely by name; describe more complex techniques in the Methods section.*
☒	☐	A description of all covariates tested
☒	☐	A description of any assumptions or corrections, such as tests of normality and adjustment for multiple comparisons
☐	☒	A full description of the statistical parameters including central tendency (e.g. means) or other basic estimates (e.g. regression coefficient) AND variation (e.g. standard deviation) or associated estimates of uncertainty (e.g. confidence intervals)
☒	☐	For null hypothesis testing, the test statistic (e.g. F, t, r) with confidence intervals, effect sizes, degrees of freedom and P value noted<br>*Give P values as exact values whenever suitable.*
☒	☐	For Bayesian analysis, information on the choice of priors and Markov chain Monte Carlo settings
☒	☐	For hierarchical and complex designs, identification of the appropriate level for tests and full reporting of outcomes
☒	☐	Estimates of effect sizes (e.g. Cohen's d, Pearson's r), indicating how they were calculated

Our web collection on statistics for biologists contains articles on many of the points above.

Software and code

Policy information about availability of computer code

Data collection	Source data was processed with R 4.0.4, SIRIUS 4.4.29 and GNPS workflow METABOLOMICS-SNETS-V2 release 26. free SMILES was processed in Java using the CDK toolkit (version 2.3) and SIRIUS libraries (version 1.4.3-SNAPSHOT).
Data analysis	Results were generated and processed with Keras / Tensorflow, version 2.4.1 on Python 3.7.10, with associated packages: matplotlib 3.3.4, pyteomics 4.4.1, rdkit 2020.09.1.0 and 2021.03.1, scipy 1.6.1, sqlite3 3.35.2, tqdm 4.59.0, dill 0.3.3, h5py 2.10.0, jpype1 1.2.1, numpy 1.19.2, pandas 1.2.3, requests 2.25.1, selfies 1.0.3, spectrum_utils 0.3.4, tensorflow 2.4.1, bitstring 3.1.7, chempy 0.8.0, pywebio 1.3.3, molmass 2020.6.10, pyyaml 5.4.1; based on Miniconda 4.10.3, with additional Java libraries BitToBoolean (edu.rutgers.sakai.java.util.BitToBoolean, no version) and ProgressBar (me.tongfei.progressbar 0.8.1), and the shell utility yq (4.9.6). Java code was compiled with Maven 3.7.0 for OpenJDK 11<br>Results were post-processed, analyzed and plotted with R 4.0.4 and 4.1.1 with packages colorblindr (https://github.com/clauswilke/colorblindr) 0.1.0@e6730be, directlabels 2021.1.13, ggthemes 4.2.4, glue 1.4.2, gridExtra 2.3, khroma 1.7.0, RColorBrewer 1.1-2, scales 1.1.1, tidyverse 1.3.1. Mass spectra library search and interpretation was conducted with NIST 2020 (MS Search 2.4, MS Interpreter 3.4.4). The container was built and tested on Docker 19.03.6, Ubuntu 18.04.4 LTS, with 16 GB RAM; Docker 19.03.8 on Ubuntu 20.04.2 LTS, with 32 GB RAM; Docker Desktop 2.3.0.4 (46911; engine 19.03.12) on Windows 10.0.10942 with 16 GB RAM; and Docker Desktop 4.1.1 (engine v20.10.8) on Windows 10 20H2 (19042.2037).<br>The entire data and code for reproducing the analysis and and plots is available on Zenodo, https://zenodo.org/record/5705830, and Github, https://github.com/meowcat/MSNovelist.

For manuscripts utilizing custom algorithms or software that are central to the research but not yet described in published literature, software must be made available to editors and reviewers. We strongly encourage code deposition in a community repository (e.g. GitHub). See the Nature Portfolio guidelines for submitting code & software for further information.

Data

Policy information about [availability of data](#)

All manuscripts must include a [data availability statement](#). This statement should provide the following information, where applicable:

- Accession codes, unique identifiers, or web links for publicly available datasets
- A description of any restrictions on data availability
- For clinical datasets or third party data, please ensure that the statement adheres to our [policy](#)

The dataset and scripts required to reproduce the validation, bryophyte analysis, and figures are provided on Zenodo, doi: 10.5281/zenodo.5705830. The dataset MTBLS709 analysed during the current study is available in the MetaboLights repository, https://www.ebi.ac.uk/metabolights/MTBLS709. Processed data is available on the GNPS repository, https://gnps.ucsd.edu/ProteoSAFe/status.jsp?task=b8b481147b844ebda2481bf9656baec8. The HMDB and COCONUT databases are available on Zenodo, https://zenodo.org/record/3375500 and https://zenodo.org/record/3778405. The DSSTox database is available at ftp://newftp.epa.gov/COMPTOX/Sustainable_Chemistry_Data/Chemistry_Dashboard/MetFrag_metadata_files/CompTox_17March2019_SelectMetaData.csv.

Field-specific reporting

Please select the one below that is the best fit for your research. If you are not sure, read the appropriate sections before making your selection.

☒ Life sciences ☐ Behavioural & social sciences ☐ Ecological, evolutionary & environmental sciences

For a reference copy of the document with all sections, see [nature.com/documents/nr-reporting-summary-flat.pdf](#)

Life sciences study design

All studies must disclose on these points even when the disclosure is negative.

Sample size	The existing datasets GNPS (n=3863) and CASMI (n=127 positive mode spectra) were used for evaluation. This corresponds to the entire dataset available, minus five instances from GNPS that could not be processed.
Data exclusions	n=5 structures from dataset GNPS (originally n=3868) were not correctly parsed and excluded from the evaluation set.
Replication	Analysis and evaluation was performed with batch processing scripts and can be replicated, the entire analysis pipeline is available from Zenodo and Github.
Randomization	Evaluation data (GNPS and CASMI) was split into ten folds in a structure-disjoint manner: An InChIKey hash, representing a unique structure, was computed for all input SMILES strings, and all unique InChIKeys were randomized into ten folds. Exception: All InChIKeys present in the CASMI dataset were assigned to the same fold (0), such that the complete dataset was unknown to the corresponding model. Each SMILES was then assigned to the fold corresponding to its InChIKey.
Blinding	Blinding was not relevant, as the splits and evaluation were performed and verified computationally. To verify that structure-disjoint splitting worked correctly, the structure-disjoint splitting and absence of test data in the training set was additionally verified manually for some instances.

Reporting for specific materials, systems and methods

We require information from authors about some types of materials, experimental systems and methods used in many studies. Here, indicate whether each material, system or method listed is relevant to your study. If you are not sure if a list item applies to your research, read the appropriate section before selecting a response.

Materials & experimental systems

n/a	Involved in the study	
☒	☐	Antibodies
☒	☐	Eukaryotic cell lines
☒	☐	Palaeontology and archaeology
☒	☐	Animals and other organisms
☒	☐	Human research participants
☒	☐	Clinical data
☒	☐	Dual use research of concern

Methods

n/a	Involved in the study	
☒	☐	ChIP-seq
☒	☐	Flow cytometry
☒	☐	MRI-based neuroimaging
